# How a gene fuels ear infections

**DOI:** 10.7554/eLife.105612

**Published:** 2025-01-22

**Authors:** Sedigheh Delmaghani, Aziz El-Amraoui

**Affiliations:** 1 Université Paris Cité, Institut Pasteur, AP-HP, Inserm, CNRS, Fondation Pour l'Audition, Institut de l’Audition, IHU reconnect, Progressive Sensory Disorders, Pathophysiology and Therapy Unit Paris France

**Keywords:** down syndrome, otitis media, DYRK1A, inflammation, hearing loss, Middle ear, Mouse, Human samples

## Abstract

The DYRK1A enzyme is a pivotal contributor to frequent and severe episodes of otitis media in Down syndrome, positioning it as a promising target for therapeutic interventions.

**Related research article** Tateossian H, Southern A, Vikhe P, Lana-Elola E, Watson-Scales S, Gibbins D, Williams D, Purnell T, Mburu P, Parker A, Norris DP, Santos-Cortez RLP, Herrmann BW, Wells S, Lad HV, Fisher EM, Tybulewicz VL, Brown SD. 2025. DYRK1A kinase triplication is the major cause of otitis media in Down syndrome. *eLife* **14**:RP101969. doi: 10.7554/eLife.101969.

Otitis media with effusion, or ‘glue ear’, occurs when the middle ear becomes inflamed or infected and fills up with fluid – often due to bacteria or viruses migrating from the nasal passages to the ear through the Eustachian tube (Nokso-Koivisto et al., 2024). While this pathology is one of the most common causes of hearing loss worldwide, in most children, episodes of otitis media are temporary and resolve with treatment.

For individuals with Down syndrome, however, otitis media is both more frequent and more severe, often causing lasting damage to the delicate structures of the ear (Manickam et al., 2016; Maris et al., 2014). Studies suggest that by age five, nearly 70% of children with Down syndrome will have developed the condition, with many requiring multiple surgical interventions to alleviate it (Manickam et al., 2016; Maris et al., 2014). The resulting chronic hearing impairment significantly hinders communication and daily functioning, highlighting the need for targeted interventions to improve quality of life. Yet why this condition disproportionately affects those with Down syndrome has remained poorly understood (Porter et al., 2023). Now, in eLife, Steve Brown, Hilda Tateossian and colleagues have identified a gene known as *DYRK1A* as a critical driver of otitis media in Down syndrome (Tateossian et al., 2025).

The researchers, who are based at the MRC Harwell Institute, the Francis Crick Institute and other institutes in the UK and US, first used several advanced mouse models which, together, replicate the genetic profile observed in Down syndrome. In humans, the condition is due to the presence of a full or partial extra copy of chromosome 21, often resulting in the overexpression of proteins coded by these multiple triplicated genes. In mice, the sequences equivalent to the genes on human chromosome 21 are spread across a number of mouse chromosomes, including chromosomes 10, 16, 17 (Figure 1A). Examining which type of mice model presented symptoms of otitis media and hearing loss revealed that the condition was associated with the presence of an extra copy of genes on mouse chromosome 16; in particular, further analyses identified a link with a shorter region harboring 12 genes, including one known as *DYRK1A* (Figure 1B). This sequence, which is present on human chromosome 21, encodes an enzyme involved in various signaling pathways important for development and immunity. Its implication in cognitive disabilities, Alzheimer’s disease and congenital heart defects, for example, has been well-documented (Murphy et al., 2024; Lana-Elola et al., 2024). Reducing the amount of DYRK1A expressed in the mouse models restored middle ear health, convincingly linking *DYRK1A* to the development of otitis media (Tateossian et al., 2025).

**Figure 1.**
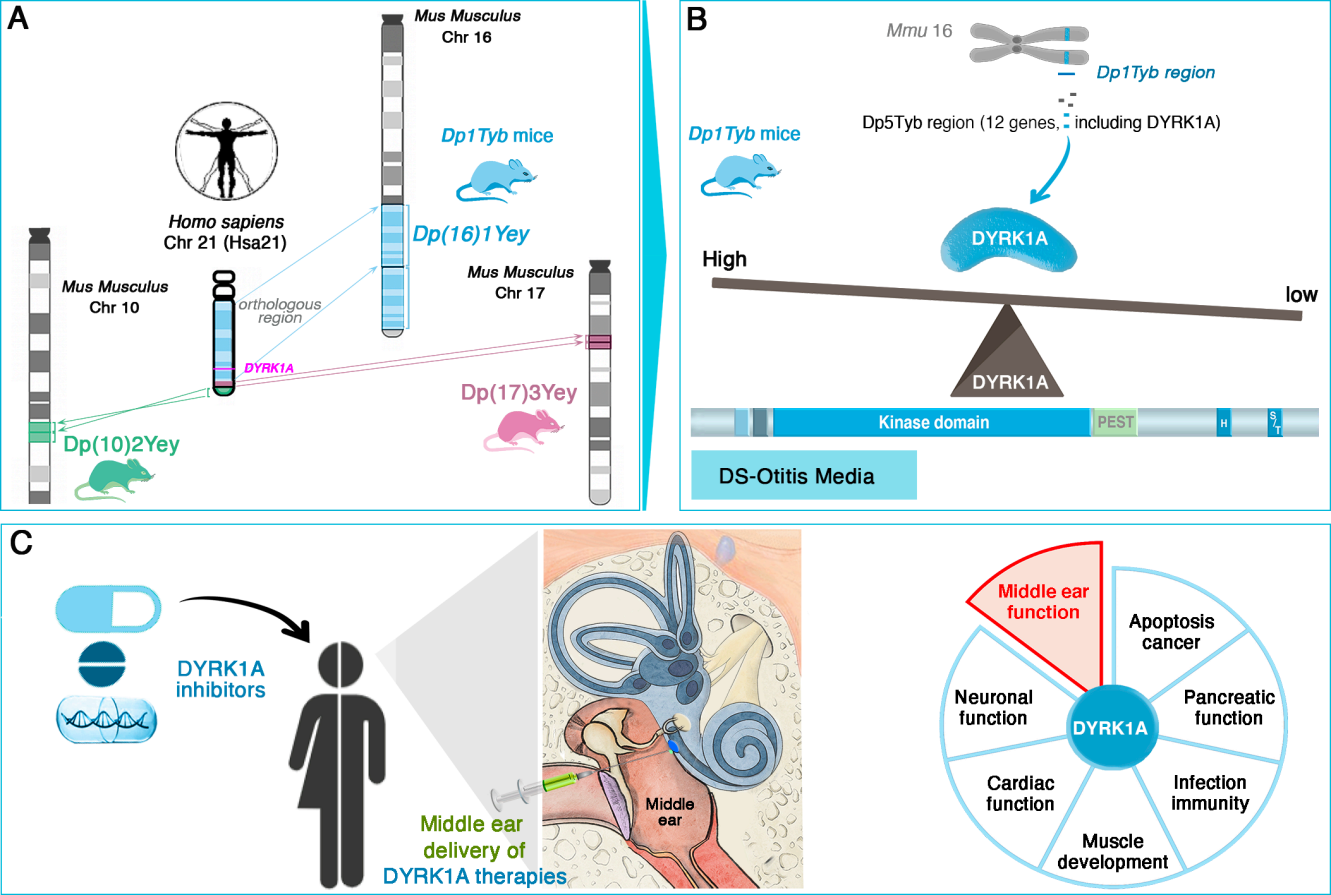
*DYRK1A* as a key driver and therapeutic target in Down syndrome-related otitis media. (**A**) Mouse models with extra copies of genes on chromosomes 10 (green), 16 (blue), and 17 (pink) mimic the genetic changes seen in human trisomy 21. Experiments conducted on these animals enabled the identification of the Dp(16)1Tyb region (located on mouse chromosome 16, Mmu16) as the genetic driver of Down syndrome-related otitis media. This region carries a gene (bright pink) coding for the DYRK1A protein. (**B**) Further investigations into the Dp1Tyb region (blue) on mouse chromosome 16 allowed Tateossian et al. to refine the locus associated with increased otitis media to a shorter region harboring 12 genes, including *DYRK1A*. Mice in which this region is present in three copies overexpress DYRK1A (blue shape): this enzyme (which includes a kinase and various other domains, including PEST and S/T domains) is an enzyme important for various signaing pathways. DYRK1A overexpression leads to pathological changes that create a middle ear environment prone to persistent infections and effusions. (**C**) DYRK1A inhibitors (blue), including small molecules and natural products, emerge as promising therapeutic agents. Their localized delivery to the middle ear offers a targeted approach to mitigate otitis media; they may also potentially address broader Down syndrome-related health deficits in which *DYRK1A* may be involved, such as deficits in muscle development or cardiac function.

Image credit: Middle ear illustration adapted from Figure 4 of Delmaghani and El-Amraoui, 2020 (CC BY 4.0).

Next, Tateossian et al. conducted experiments to uncover how the overexpression of *DYRK1A* was linked to ear health, revealing several interconnected mechanisms. Indeed, elevated levels of DYRK1A were associated with a rise in proinflammatory molecules, known as IL-6 and IL-17, in the fluid present in the middle ear, potentially contributing to sustain chronic inflammation. Mutants also showed upregulated VEGF signaling which, in turn increased the permeability of blood vessels – a process that can result in leakage and therefore exacerbate inflammation and fluid build-up. Finally, electron microscopy approaches revealed that mice overexpressing *DYRK1A* experienced significant loss of the small hairs tasked with clearing fluids and pathogens in the middle ear. Together, these mechanisms create an environment highly susceptible to infections and fluid accumulation.

To validate these findings in humans, the team analyzed clinical samples from children with Down syndrome. Among the 12 genes studied within the implicated region, *DYRK1A* was the most significantly overexpressed compared to controls, further supporting its role as a central driver of otitis media in Down syndrome. This alignment between animal models and human data highlights the robustness of the study, as well as its potential clinical relevance.

*DYRK1A* is indeed a promising therapeutic target, with the recent development of DYRK1A inhibitors, such as leucettinib-21 and aristolactam BIII, offering many opportunities for targeted interventions (Kay et al., 2016; Murphy et al., 2024). The accessibility of the middle ear for localized drug delivery makes this a particularly attractive approach (Figure 1C; Delmaghani and El-Amraoui, 2020). Future research should prioritize investigating the safety and efficacy of DYRK1A inhibitors in preclinical and clinical trials. Longitudinal studies in individuals with Down syndrome could also assess whether modulating *DYRK1A* expression confers broader health benefits, such as improvements in cognitive function, cardiac health or immune regulation.

Despite these breakthroughs, several questions remain unanswered. For instance, how do other genes within the implicated region interact with *DYRK1A* to influence the likelihood of developing otitis media? And to what extent are these genetic effects modulated by environmental factors, such as early-life infections or differences in the communities of microbes present in the ear and respiratory tract (Elling et al., 2023)? By starting to shed light on the genetic and biological mechanisms driving otitis media in Down syndrome, the work by Tateossian et al. enables future research studies that hold the potential to transform health outcomes and improve quality of life.
